# Hepatitis C prevalences in the psychiatric setting: Cost-effectiveness of scaling-up screening and direct-acting antiviral therapy

**DOI:** 10.1016/j.jhepr.2021.100279

**Published:** 2021-03-18

**Authors:** François Girardin, Chris Painter, Natalie Hearmon, Lucy Eddowes, Stefan Kaiser, Francesco Negro, Nathalie Vernaz

**Affiliations:** 1 Division of Clinical Pharmacology and Toxicology, Department of Anaesthesiology, Clinical Pharmacology, Intensive Care and Emergency Medicine, Geneva University Hospitals, Geneva, Switzerland; 2 Service of Clinical Pharmacology, Department of Laboratory Medicine and Pathology, Lausanne University Hospital (CHUV) and University of Lausanne, Lausanne, Switzerland; 3 Costello Medical, London, UK; 4 Division of Adult Psychiatry, Geneva University Hospitals, Geneva, Switzerland; 5 Divisions of Gastroenterology and Hepatology and of Clinical Pathology, Geneva University Hospitals, Geneva, Switzerland

**Keywords:** Hepatitis C infection, Screening strategy, Direct-acting antiviral agents, Cost-effectiveness model, Psychiatric disorder, DAA, direct-acting antiviral, ICER, Incremental cost-effectiveness ratio, NMB, net monetary benefit, PMI, patients with mental illnesses, PSA, probabilistic sensitivity analysis, QALY, quality-adjusted life-year, WTP, willingness-to-pay

## Abstract

**Background & Aims:**

Patients hospitalised because of mental illness often have risk factors for contracting HCV. Scaling-up HCV screening for all psychiatric inpatients as a case-detection strategy for viral elimination is underexplored. This study aimed to evaluate the cost-effectiveness of scaling-up HCV screening and treatment for psychiatry hospital admissions in Switzerland *vs.* the current standard-of-care risk-based approach, where only those with a history of substance misuse disorder are offered testing.

**Methods:**

HCV prevalence by history of substance misuse disorder was analysed in medical records from inpatient admissions to a Swiss psychiatry department. Cost-effectiveness was analysed from a healthcare provider perspective through a decision-tree screening model, using these HCV prevalence data. Model and parameter uncertainty were assessed using deterministic and probabilistic sensitivity analyses.

**Results:**

Prevalence of HCV in psychiatry inpatients with a history of substance misuse disorder (n = 1,013) was 25.7%, compared with 3.5% among the remaining inpatients (n = 3,535). Scaling up HCV screening and treatment for all psychiatry admissions was cost-effective *vs.* the risk-based approach, with an incremental cost-effectiveness ratio of US$9,188 per quality-adjusted life-year gained. The incremental cost-effectiveness ratio remained cost-effective considering a HCV prevalence as low as 0.07%. The population-level net monetary benefit of the generalised screening approach was US$435,156,348, with 917 additional patients per year detected and treated at a cost of US$3,294 per person (*vs.* US$2,122 under risk-based screening).

**Conclusions:**

Scaling up HCV screening and treatment at diagnosis with all-oral, interferon-free regimens as a generalised approach for psychiatric admissions was cost-effective and could support reaching World Health Organization targets for HCV elimination by 2030.

**Lay summary:**

Patients hospitalised because of mental illness often have risk factors for HCV. We found that testing all psychiatry patients in hospital for HCV was cost-effective compared with testing only patients who have a history of substance misuse. Scaling up HCV testing and treatment could help to wipe out HCV.

## Highlights

• Screening all psychiatry admissions for HCV was cost-effective *vs.* standard-of-care.
• Generalised HCV screening enabled 917 extra patients to be treated per year.
• Scaling-up HCV testing to all psychiatry admissions would support HCV elimination.

## Graphical abstract

**Figure:**
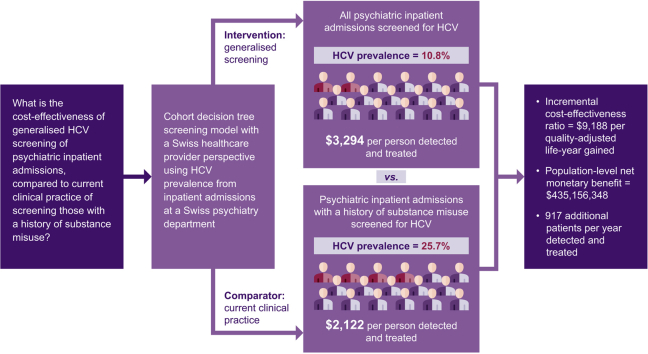

## Introduction

Neuropsychiatric disorders contribute an estimated 13% to the global burden of disease and are associated with increased physical morbidity.^1^ Patients with mental illnesses (PMI), particularly ‘severe’ disorders, are more likely to engage in behaviours that are risk factors for communicable and non-communicable disease, and might be underserved by healthcare services.^1^ Integrating services for mental and physical health can ensure more equitable access to care and reduce health inequalities.^2^

In Switzerland, between 30% and 50% of patients with severe mental illness are estimated to have a substance misuse disorder, placing them at risk of drug-related adverse events and communicable diseases, such as hepatitis C virus (HCV).^3^ Other factors associated with mental illness, such as risky sexual behaviours and incarceration, could also increase the risk of HCV infection in PMI.^4^ This is reflected in global HCV prevalence estimates of 4.6–17.4% for those with severe mental illness,^4^ compared with ~0.5–2.3% in the global population.^5^

In 2016, the World Health Organization (WHO) outlined Global Health Sector Strategy targets to guide HCV elimination by 2030, where nation states are required to diagnose 90% of prevalent cases and treat 80% of those diagnosed.^6^ A key development to support this goal is the increasing availability of highly effective, all-oral, interferon-free, direct-acting antivirals (DAAs) for HCV.^7^ DAAs allow infection to be cured in most individuals who engage with healthcare services and adhere to treatment.^7^^,^^8^ Although some countries are implementing one-time universal screening for HCV,^9^ not all countries are doing so, including high-income countries, such as Switzerland; in 2019, the Swiss Federal Office of Public Health decided to continue to prioritise at-risk groups only.^10^ Therefore, innovative case-detection strategies targeting high-risk and underserved populations, such as PMI, are still essential for driving viral elimination.^6^

By screening for HCV infection in patients engaging with psychiatric health services, high-risk individuals can be targeted, cases identified and patients linked to short-duration DAA-based treatments that are recommended for managing the physical health of PMI.^1^^,^^4^ However, it is common practice to only offer HCV testing to individuals with known risk factors for infection (*i.e.* a history of substance misuse disorder) on psychiatric hospital admission. The epidemiology of HCV among PMI is also not well characterised, preventing health authorities from accurately considering the relative costs and benefits of different approaches to diagnosing infection among PMI. Consequently, clinical and economic data are required to assess the available options and to ensure the optimal use of healthcare resources.^4^

This study explored the prevalence of HCV infection in patients admitted to a large psychiatric hospital department. Using real-world prevalence estimates from individual patient data, the study assessed the cost-effectiveness of a generalised screening approach, where all patients were offered an HCV test on psychiatric hospital admission, compared with a risk-based screening approach, where only those with a history of substance misuse disorder were offered a test. This risk-based approach is currently used in hospitals in many countries.

## Patients and methods

The methods used for the epidemiological component of this study are discussed first, followed by the modelling approach. Data from the epidemiological investigation provided inputs for the cost-effectiveness evaluation.

### Epidemiological study

#### Study design, population and outcomes

Epidemiological data on HCV infection in patients admitted to a psychiatric unit were acquired from a retrospective review of clinical records at the University Hospitals of Geneva (Hôpitaux Universitaires de Genève; HUG), the largest university hospital in Switzerland with specialised psychiatric inpatient and outpatient facilities. Adult patients eligible for inclusion were those who had been hospitalised in the HUG Psychiatry Department from January 2016 until July 2019, screened from 1990 onwards, either for antibodies to HCV (anti-HCV) or for HCV ribonucleic acid (RNA), and whose admission was coded as a mental illness (according to the ICD-10, mental and behavioural disorders [F] categories).

Patients with a history of substance misuse disorder were identified using text data mining by searching for terms such as ‘heroin’, ‘methadone’ or ‘cocaine’ in their electronic patient records. HCV prevalence was then calculated for: (i) all patients; (ii) patients without evidence of a substance misuse disorder in their medical records; and (iii) those with a history of substance misuse disorder. For the prevalence estimates, patients were deemed to be HCV positive if they were either anti-HCV antibody positive and/or HCV RNA positive.

#### Ethics

This study was performed in accordance with the Declaration of Helsinki. Ethical approval was granted by Commission cantonale d’éthique de la recherche, formerly Commission d’éthique du département de psychiatrie - approval: 09-180R. Adult participant consent was not required because anonymised data were initially aggregated or came from national and institutional registries.

#### Cost-effectiveness study

The cost-effectiveness of a comprehensive HCV screening programme for all PMI admitted to Swiss psychiatric units, compared with the current risk-based approach, was assessed by adapting a previously published economic model.^11^^,^^12^ Briefly, the model simulated the screening process in this setting and used epidemiological inputs from the retrospective review of HUG clinical records to ensure that the model was reflective of Swiss psychiatric patients. The model provided estimates of the costs and effectiveness, in terms of quality-adjusted life-years (QALYs), such that cost-effectiveness measures could be calculated. The model methodology is described further here.

#### Model structure and perspective

**Fig. 1:**
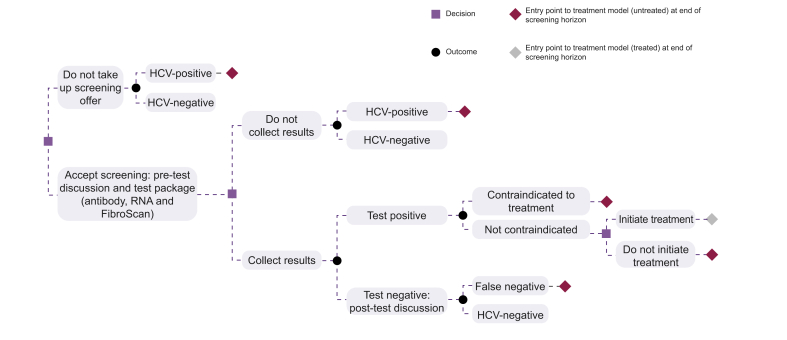
Decision tree structure of the model. HCV, hepatitis C virus; RNA, ribonucleic acid.

A previously published cohort decision tree screening model was adapted to analyse the cost-effectiveness of diagnosis and treatment, after scaling up HCV screening in PMI compared with current standard-of-care.^11^^,^^12^ The decision tree simulated the patient pathway from screening to diagnosis and treatment initiation (Fig. 1). The proportion of patients within each branch of the decision tree was estimated using uptake and outcome probabilities, representing the likelihood of attrition at each stage of the patient pathway. Costs and QALYs were assigned to each pathway.

The model took the perspective of the national healthcare provider, based on nationwide guidance from the Federal Office of Public Health.^13^ The model simulated one-off testing upon admission, whereas the costs and QALYs associated with treatment took a lifetime time horizon because of the lifelong nature of chronic HCV. Regular annual screening over a longer time period was not appropriate, given the closed nature of the inpatient population where, based on point-of-care data from the HUG Psychiatry Department, uptake of testing and of treatment was 89% and 84%, respectively. In addition, the average length of stay in Swiss inpatient facilities is usually short, limiting the potential for repeat testing; the most recent figures available for the HUG showed that the mean length of stay for psychiatric inpatients was 47 days in 2017 (median 15 days).^14^

#### Target population and screening strategies

The population size considered for the model was 60,378 individuals, the number of patients hospitalised because of mental illness in Switzerland in 2018, the most recent estimate available for the country.^15^ It was assumed that the eligible screening inpatient population was this entire PMI population, irrespective of viraemic status or awareness of HCV status.

The intervention explored was a comprehensive screening initiative to scale up HCV treatment for all inpatients in psychiatric units. This generalised strategy was compared with the current standard-of-care risk-based approach to HCV testing, implemented by psychiatric departments, whereby patients with a history of substance misuse disorder are primarily offered testing. All patients were assumed to receive a 20-min appointment to be offered screening, regardless of whether they accepted the offer. Patients who accepted the test offer were assumed to receive a further 5-min pretest discussion with a specialist. The costs associated with time spent communicating the results of the test were also included for patients who collected their results (5 min for a HCV-negative result and 20 min for a HCV-positive result). The standard test package (third-generation ELISA and quantitative HCV RNA test by RT-PCR) and a standard, all-oral, interferon-free DAA regimen were assumed for the screening and treatment approaches, respectively. Viral genotyping was not included in the test package because it is not an absolute requirement for treatment because of the availability of pan-genotypic regimens and in line with European Association for the Study of the Liver guidelines.^16^

Based on point-of-care data from psychiatric inpatients at the HUG, it was assumed that 11% of patients would decline screening, that testing would have a combined sensitivity/specificity of 1, and that those receiving a positive test result would experience a negative impact on their quality of life (modelled as a disutility).^11^

The model also permitted patients found to have chronic HCV infection to decline treatment. Those who accepted treatment received the costs and QALYs associated with treatment, whereas those who did not receive treatment were assigned costs and QALYs corresponding to natural HCV disease progression.

#### Model inputs

The screening methods and model inputs were derived from individual psychiatric inpatient data. The target population was assumed to align with the demographic and epidemiological data acquired from a retrospective review of records from the HUG Psychiatry Department. The number of patients admitted to psychiatric units in Switzerland was obtained from the Swiss Health Observatory (ObSan, Federal Office of Statistics).^15^ Model inputs related to treatment effects and natural disease progression were derived from a peer-reviewed cost-effectiveness model of an all-oral, interferon-free DAA treatment regimen in people who inject intravenous drugs.^17^ The lifetime costs (including drug acquisition, resource use and monitoring costs) per treated or untreated patient with HCV were also sourced from the same model.^17^ Further model inputs were derived from targeted literature searches, publicly available databases and discussion with clinical experts. Model inputs are summarised in Table 1, Table 2.

**Table 1:** Population and clinical model inputs.

Input	Value	Source
**Population**		
Eligible population size	60,378	Unpublished 2018 data from Swiss Health Observatory
Male proportion	51.4%	HUG Psychiatry Department
Average age (years)	46.5	HUG Psychiatry Department
HCV prevalence (population currently tested)	25.7%	HUG Psychiatry Department
HCV prevalence (population not currently tested)	3.5%	HUG Psychiatry Department
Proportion who present for testing (current screening)	38.3%	HUG Psychiatry Department
**Screening and diagnosis**		
RNA PCR sensitivity	1	Assumed
RNA PCR specificity	1	Assumed
Proportion of tests in which confirmation of diagnosis is requested	3.4%	HUG audit on quality of laboratory results
Proportion of patients who receive an antibody test as part of test package	39.0%	HUG audit on quality of laboratory results
Proportion of patients who receive an RNA test as part of test package	94.9%	HUG audit on quality of laboratory results
Probably of accepting invitation to screening/testing	89.0%	Point of care, HUG Psychiatry Department
Probably of collecting test results	95.0%	Point of care, HUG Psychiatry Department
Probably of initiating treatment	84.0%	Point of care, HUG Psychiatry Department
Disutility of HCV-positive result	0.02	Based on estimate by Singer and Younossi^28^ (from Rodger *et al.* 1999)^29^
**Treatment**		
Lifetime QALYs per infected person (no antiviral treatment)	16.5	Scott *et al.* 2016^18^
Lifetime QALYs per infected person (early treatment)	21.7	Scott *et al.* 2016^18^

HCV, hepatitis C virus; HUG, University Hospitals of Geneva; PCR, polymerase chain reaction; QALY, quality-adjusted life-year; RNA, ribonucleic acid.

**Table 2:** Cost model inputs.

Input	Value	Source
**Screening**		
Test offer and voluntary counselling cost (regardless of uptake); 20-min consultation	CHF 62.53	TarMed Suisse. Tariff version: 1.09, from Jan 1, 2018. TARMED CT00.0010 + CT00.0020 + CT00.0030
Pretest discussion cost; 5-min consultation	CHF 17.86	TarMed Suisse. Tariff version: 1.09, from Jan 1, 2018. TARMED CT00.0050
Cost of communicating results, HCV viraemia negative; 5-min consultation	CHF 17.86	TarMed Suisse. Tariff version: 1.09, from Jan 1, 2018. TARMED CT00.0050
Cost of communicating results, HCV viraemia positive; 20-min consultation	CHF 62.53	TarMed Suisse. Tariff version: 1.09, from Jan 1, 2018. TARMED CT00.0010 + CT00.0020 + CT00.0030
Cost of antibody test	CHF 66.00	TarMed Suisse. Tariff version: 1.09, from Jan 1, 2018. TARMED CA3070.00 (HCV, Ig or IgG, test)
Cost of RNA test (quantitative PCR for HCV)	CHF 180.00	TarMed Suisse. Tariff version: 1.09, from Jan 1, 2018. TARMED CA3073.00 (HCV, RNA amplification)
Cost of FibroScan (including consultation)	CHF 183.55	TarMed Suisse. Tariff version: 1.09, from Jan 1, 2018. TARMED CT00.0056 + CT00.0161 + CT39.3515 + CT39.3660 + CT00.2285
Confirmation of diagnosis if HCV positive	CHF 180.00	TarMed Suisse. Tariff version: 1.09, from Jan 1, 2018. TARMED CA3073.00
**Treatment, including drug acquisition, monitoring and resource use**		
Lifetime cost per infected person (no antiviral treatment)	CHF 15,970	Scott *et al.* 2016^3^
Lifetime cost per infected person (early treatment)	CHF 55,336	Scott *et al.* 2016^3^

CHF, Swiss francs; HCV, hepatitis C virus; HUG, University Hospitals of Geneva; PCR, polymerase chain reaction; RNA, ribonucleic acid.

Given that the model explored a single screening round, discounting of costs and QALYs was not applied. The model from which the lifetime treatment costs and QALYs were sourced applied 3% discounting to costs and QALYs.^17^

#### Model outputs

The primary outputs from the screening model were combined with the treatment model outputs to calculate the incremental cost-effectiveness ratio (ICER) of generalised *vs.* risk-based screening, with benefits given in QALYs and costs in Swiss francs, assumed to be equivalent to US dollars (as per exchange rates in November 2020). A willingness-to-pay (WTP) threshold, which is the valuation of the health benefit in monetary terms, of US$100,000/QALY was chosen because it falls within the range of recommended WTP thresholds for cost-effectiveness analyses for Switzerland.^18^^,^^19^ A lower WTP threshold of US$50,000 per QALY gained was also considered.

The model produced further standard cost-effectiveness outputs, such as the net monetary benefit (NMB). The NMB is defined as the incremental effects multiplied by the WTP threshold of US$100,000/QALY, minus the incremental costs. Thus, a positive NMB indicates that the intervention (generalised screening) is cost-effective at each given WTP threshold. Further outputs included separate clinical- and cost-focussed results, such as the cost of diagnosis per HCV patient identified (both completing the diagnosis process and initiating treatment).

#### Analyses

A deterministic sensitivity analysis was performed to identify which inputs influenced the ICER most, in which each input was varied by 20% above and below the base-case value. Parameters were ranked hierarchically by their impact on the ICER.

A probabilistic sensitivity analysis (PSA) was performed from 1,000 Monte-Carlo simulations, to test the robustness of model outputs. A standard deviation of 20% of the mean was used to derive the probability distributions for the PSA. The PSA results were assessed on the cost-effectiveness plane using a scatter plot of incremental costs *vs.* incremental QALYs for the generalised HCV screening approach *vs.* the current risk-based strategy. The probability of cost-effectiveness of the generalised screening approach was tested at a range of WTP thresholds to generate a cost-effectiveness acceptability curve. This provided an overall probability of cost-effectiveness at the chosen WTP threshold of US$100,000/QALY.

## Results

### Prevalence estimates

The HCV prevalence study comprised 5,420 inpatients, of whom 4,548 were eligible for inclusion. Patients were excluded if they were a paediatric admission (n = 581), lacked a relevant ICD-10 diagnostic code (n = 279) or were the child of an admitted patient (n = 12). During the 4-year study period (2016–2020), 32.0% of inpatients were rehospitalised in the psychiatric department.

The proportion of males in the cohort was 51.4% (males aged 18–96; females aged 16–100) and the average age of the cohort was 46.5 years. According to the ICD-10 codes, the most common diagnoses were mood (affective) disorders (n = 1,649), schizophrenia, schizotypal and delusional disorders (n = 1,079), neurotic, stress-related and somatoform disorders (n = 679), personality disorders (n = 570), and disorders resulting from active psychoactive substance use (n = 511). Of the inpatients, 1,013 (22.3%) had a history of substance misuse disorder and their mean age was 39 years [female: 41 years (age: 16–89); male: 38 years (18–88)]. A history of cirrhosis was evident in 5.8% of inpatients with a history of substance misuse disorder compared with 2.4% of those without.

**Fig. 2:**
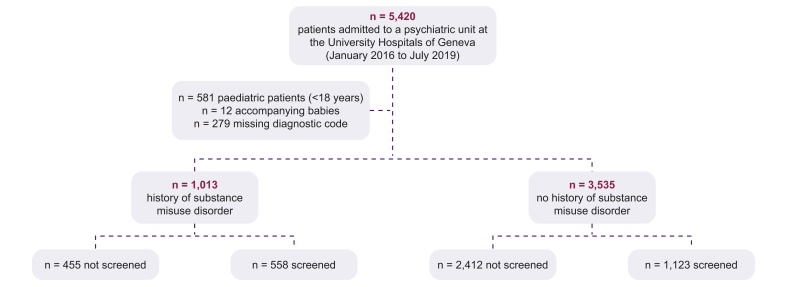
Patient flow diagram of retrospective study eligibility and HCV screening status. HCV, hepatitis C virus; RNA, ribonucleic acid.

HCV screening was performed in 55.1% of inpatients with a history of substance misuse disorder compared with 31.8% of those without (Fig. 2). HCV prevalence in inpatients with a history of substance misuse disorder (positive for anti-HCV antibodies or HCV RNA) was 25.7% (117/155 patients were viraemic). For those patients without a history of substance misuse disorder, HCV prevalence was 3.5% (28/43 patients were viraemic), resulting in an overall prevalence of 10.8% for all admissions.

### Base case

Generalised screening of all admitted PMI was cost-effective compared with risk-based screening, with a base-case ICER of US$9,188 per QALY (Table 3), much lower than the WTP thresholds of US$50,000 and US$100,000 per QALY gained.^18^^,^^19^ At the population level, generalised screening was associated with a greater overall cost (incremental costs: US$44,028,136) and more QALYs (incremental QALY gain: 4,792), equivalent to US$729 and 0.08 QALYs per person. The NMB of generalised screening was US$435,156,348 for the whole target population.

**Table 3:** Summary of model results.

	Generalised screening approach	Current risk-based screening approach
Number of patients initiating treatment	5,108	4,191
Total costs	US$333,593,535	US$289,565,399
Total QALYs	1,213,151	1,208,359
Cost per person	US$5,525	US$4,796
QALYs per person	3.07	2.99
Incremental cost of generalised screening approach	US$44,028,136	
Incremental QALYs gained from generalised screening approach	4,792	
Incremental cost of generalised screening approach per person	US$729	
Incremental QALYs gained from generalised screening approach per person	0.08	
ICER	US$9,188 per QALY	

ICER, incremental cost-effectiveness ratio; QALY, quality-adjusted life-year.

The total cost of screening per person completing the diagnosis pathway was lower for generalised screening compared with the current risk-based approach (US$330 *vs.* US$455). The total cost of screening per HCV-positive patient initiating treatment with DAAs was US$2,122 and US$3,294 in the current risk-based approach and the generalised screening approach, respectively.

The number of individuals treated increased from 4,191 to 5,108 with the generalised screening programme, with 1,099 additional patients with HCV linked to care and 917 additional patients with HCV initiating treatment in the generalised approach. The number of patients that were needed to be screened to link one patient with HCV to HCV care was higher for the generalised approach compared with the current risk-based approach (10.0 *vs.* 4.7 people tested per HCV-positive diagnosis), given the lower HCV prevalence in the overall patient population compared with those with a history of substance misuse disorder.

### Sensitivity analyses and scenario analyses

**Fig. 3:**
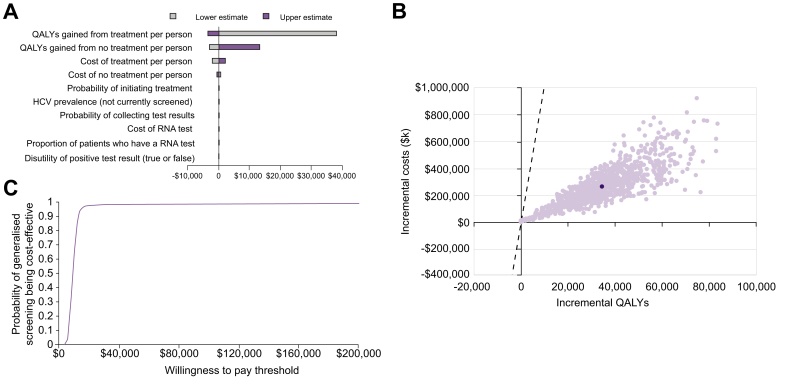
Model sensitivity analyses results. (A) Tornado plot of the deterministic sensitivity analysis. (B) Scatter plot of the probabilistic sensitivity analysis. (C) Cost-effectiveness acceptability curve. Light purple dots: individual simulations. Dark-purple dot: mean of all simulations. HCV, hepatitis C virus; QALY, quality-adjusted life year; RNA, ribonucleic acid.

The deterministic sensitivity analysis identified the inputs with the greatest influence on the ICER (the key drivers of cost-effectiveness) as: QALYs gained from treatment/no treatment; cost of treatment/no treatment; probability of initiating treatment; and HCV prevalence in the subgroup of patients not currently offered screening (Fig. 3A). All resulting ICERs were well below the WTP threshold of US$100,000/QALY.

The results of the PSA showed that 98.2% of simulations predicted generalised screening of patients admitted to psychiatric units to be cost-effective (WTP threshold of US$100,000/QALY; Fig. 3B). The probability of generalised screening being cost-effective compared with current risk-based HCV screening remained stable on the cost-effectiveness acceptability curve when the WTP threshold was varied (Fig. 3C).

**Fig. 4:**
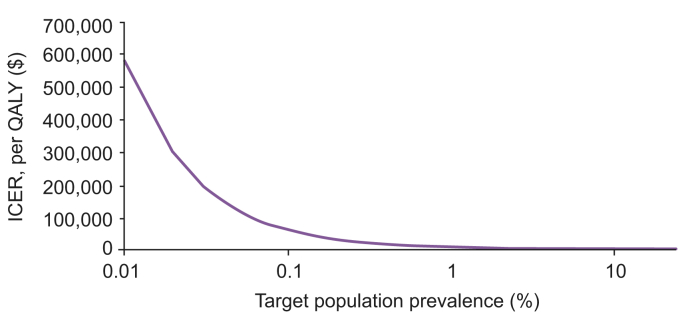
Impact of target population prevalence on the ICER. ICER, incremental cost-effectiveness ratio.

The most recent estimate of the prevalence of HCV from the Swiss Federal Office of Public Health suggests a prevalence of 0.45%–0.54% in the Swiss population.^20^ Assuming a target population prevalence of 0.5% in the model, the ICER increased to US$19,026 per QALY, suggesting that a generalised approach to screening and treating is cost-effective, regardless of whether HCV prevalence in those not currently offered screening on admission to a psychiatry department approaches that of the general population in Switzerland (Fig. 4).

At a WTP threshold of US$100,000/QALY, the NMB remained positive with a target population prevalence as low as 0.07% (population NMB: US$1,002,650; data not shown), indicating that scaling up screening was cost-effective compared with the current approach over a range of HCV prevalence.

## Discussion

As countries progress towards viral elimination by using DAAs to treat HCV infection, screening programmes focussed on underserved communities will become increasingly important.^21^ Psychiatric hospitals represent an overlooked setting, where high-risk individuals can be diagnosed and treated in a controlled environment.^1^^,^^4^

Results from this analysis demonstrated that generalised screening of all patients admitted to psychiatric hospital units, combined with treatment using oral interferon-free DAA regimens, is likely to be cost-effective compared with the current risk-based screening strategy at a range of WTP thresholds. This result is consistent with WHO guidelines for the management of physical health conditions in people with severe mental illness, which recommend screening for HCV in this population and emphasise the use of interferon-free DAA regimens because of the severe psychological adverse effects of interferon-based treatments (depression, mental confusion, anxiety and psychosis).^1^^,^^22^ Furthermore, drug–drug interactions between opioids, illicit substances or psychotropic medication and DAA regimens are limited, making DAA regimens suitable for treating this population.^23^

In Switzerland, disease elimination models suggest that 1,500 patients need to be diagnosed annually, beginning in 2020, if Global Health Sector Strategy targets are to be achieved.^24^ With 60,378 individuals treated as inpatients for mental illness in Switzerland annually and an estimated HCV prevalence of 10.8%, diagnosing and treating the 6,521 HCV-infected PMI using psychiatric hospitals and outpatient units would have a key role in meeting this target.

However, with inpatient treatment being increasingly substituted by intensive outpatient care, considering the generalisability of results of this study is important. It will be necessary to evaluate whether HCV screening can be comparably cost-effective in the outpatient setting, where linkage to care and treatment initiation rates might be lower.^25^ In addition, different countries have different clinical thresholds for hospitalisation because of mental illness, and a lower proportion of patients might be hospitalised for their mental illness in some countries.

Furthermore, the HCV prevalence estimates used in this study, acquired from the HUG Psychiatry Department, might not be representative of the true HCV prevalence of psychiatric inpatients in Switzerland or in other countries. Nevertheless, our analysis accounted for variation in these parameters and generalised screening remained cost-effective at a WTP threshold of US$100,000/QALY down to an HCV prevalence of 0·07%. A similar HCV prevalence threshold for cost-effectiveness was recently reported for universal HCV screening in pregnant women in the USA,^26^ suggesting that screening and treatment programmes can be cost-effective at low prevalence.

Integration of psychiatric inpatient and outpatient facilities into the wider network of HCV services will also be necessary to ensure reliable progression from diagnosis to cure for patients, particularly because most patients spend too short a duration in hospital to receive a complete course of treatment (typically 12 weeks); the most recent estimates from the HUG show the proportion of patients staying for more than 100 days was approximately 7.2%.^14^ Training of psychiatric staff to ensure effective implementation of HCV services will increase costs.^4^ However, this study showed that generalised screening would likely remain cost-effective, despite additional implementation costs, whereas integrating mental and physical healthcare might bring wider operational benefits for healthcare services and clinical benefits for patients.

Our results are most relevant to countries with similar HCV epidemiology, existing screening programmes and treatment availability. A systematic review and meta-analysis investigating HCV in patients with severe mental illness (out- and inpatients), reported a prevalence of 4.9% (95% CI: 3.0–7.9%) in Europe, 17.4% (95% CI: 13.2–22.6) in North America and 3.1% (95% CI: 1.0–9.3) in Oceania.^4^ However, these values were based on a small number of studies, where viral prevalence varied considerably (Europe: 0.7–10.7%; North America: 2.7–38%; Oceania: 3.1%) compared with 10.8% estimated in hospitalised PMI in this study.^4^ Although further research is needed to better characterise the epidemiology of infection within different PMI populations, our analyses suggest that expanded screening of inpatients at psychiatric departments represents a clinically beneficial and cost-effective approach for other high-income countries with similar healthcare systems.

Overall, this study supports the scaling up of screening and treatment programmes designed to eliminate HCV.^6^ Offering DAAs at the point of diagnosis is an effective method of containing disease progression in groups with a high prevalence of HCV infection, such as PMI, people living in detention or people who inject drugs.^4^^,^^11^^,^^12^ Worldwide projections also indicate that outreach screening and DAA provision at the point of diagnosis are more efficient at reducing HCV-related mortality compared with current infection control and harm reduction measures for people who inject drugs.^27^

As health authorities look to target HCV screening initiatives at underserved and high-burden populations to achieve the WHO incidence reduction target of 80% by 2030, this study provides further evidence suggesting PMI as a high-risk group and that psychiatric facilities are a crucial location in which to diagnose and treat HCV.^6^ Although further efforts are required to characterise viral epidemiology among PMI in many countries, results from this study support a generalised HCV screening approach in psychiatric hospitals.

## Financial support

This work was supported by 10.13039/100005564Gilead, the University Hospitals of Geneva and the University of Lausanne. The development of the model was funded by Gilead. Individual patient data acquisition from medical records was funded by the University Hospitals of Geneva (Department of Anaesthesiology, Clinical Pharmacology, Intensive Care, and Emergency Medicine). The funding sources did not have a role in the collection, analysis and interpretation of data; in the writing of the report; or in the decision to submit the article for publication.

## Authors’ contributions

Conceptualisation: FG, NV. Formal analysis: FG, CP, NH, LE, FN, NV. Investigation: FG, NV. Methodology: CP, NH, LE. Writing – original draft: LE. Writing – review and editing: FG, CP, NH, LE, SK, FN, NV.

## Data availability statement

The data that support the findings of this study are available from the corresponding author upon reasonable request.

## Conflicts of interest

F.G. is the representative of Swiss hospitals at the Federal Drug Commission (Federal Office of Public Health) and expert for InnoSuisse - the Federal Agency for Innovation (section Life Sciences). S.K. has received honoraria for cognitive test and training software from Schuhfried. F.N. advises Gilead, Merck and AbbVie, and has received a research grant from 10.13039/100005564Gilead. L.E. and N.H. are employees of Costello Medical, and C.P. was an employee of Costello Medical at the time of the study.

Please refer to the accompanying ICMJE disclosure forms for further details.
