# Supplementary material for: Hepatitis C prevalences in the psychiatric setting: Cost-effectiveness of scaling-up screening and direct-acting antiviral therapy
Source: JHEP Rep. 2021 Mar 18;3(3):100279. doi: 10.1016/j.jhepr.2021.100279 (PMC8424278; doi:10.1016/j.jhepr.2021.100279)
Supplement: Multimedia component 1 [file mmc1.pdf]

JHEP|Reports

JHEP Reports

CTAT methods

Tables for a "Complete, Transparent, Accurate and Timely account" (CTAT) are now mandatory for all revised submissions. The aim is to enhance the reproducibility of methods.

- Only include the parts relevant to your study
- Refer to the CTAT in the main text as 'Supplementary CTAT Table'
- Do not add subheadings
- Add as many rows as needed to include all information
- Only include one item per row

If the CTAT form is not relevant to your study, please outline the reasons why:

We believe CTAT is not relevant to our study; our study is a retrospective review of medical records and health economic model

1.1 Antibodies

Name	Citation	Supplier	Cat no.	Clone no.
N/a				

1.2 Cell lines

Name	Citation	Supplier	Cat no.	Passage no.	Authentication test method
N/a					

1.3 Organisms

Name	Citation	Supplier	Strain	Sex	Age	Overall n number
N/a						

1.4 Sequence based reagents

Name	Sequence	Supplier
N/a		

1.5 Biological samples

Description	Source	Identifier
N/a		

1.6 Deposited data

Name of repository	Identifier	Link
N/a		

1.7 Software

Software name	Manufacturer	Version
Excel	Microsoft	

1.8 Other (*e.g.* drugs, proteins, vectors etc.)

N/a		

1.9 Please provide the details of the corresponding methods author for the manuscript:

Professor François Girardin
Service of Clinical Pharmacology, Department of Laboratory Medicine and Pathology, Lausanne University Hospital (CHUV), Lausanne, Switzerland
Email: Francois.Girardin@chuv.ch
Telephone: +41 (0)21 314 42 76

2.0 Please confirm for randomised controlled trials all versions of the clinical protocol are included in the submission. These will be published online as supplementary information.

N/a
